# Long-term outcomes in acute kidney injury patients treated with renal replacement therapy who were alive at hospital discharge

**DOI:** 10.1186/cc13596

**Published:** 2014-03-17

**Authors:** V Sergoyne, W De Corte, J Vanhalst, A Dhondt, S Claus, S Oeyen, J Decruyenaere, E Hoste

**Affiliations:** 1 University of Ghent, Belgium

## Introduction

Acute kidney injury (AKI) treated with renal replacement therapy (RRT) in ICU patients is associated with high mortality, chronic kidney disease (CKD) (eGFR <60 ml/minute/1.73 m^2^, or CKD stage ≥3) and end-stage kidney disease (ESKD). Data on long-term outcomes vary among studies due to differences in age, CKD, severity of illness, RRT modality and timing of initiation of RRT. Long-term patient and kidney outcomes in AKI-RRT patients were evaluated in this study.

## Methods

A retrospective study of all consecutive treated AKI-RRT patients in a 50-bed ICU academic hospital from August 2004 to December 2012. Data were retrieved from the electronic ICU, RRT and hospital patient files. Long-term outcomes data were obtained by a telephone survey.

## Results

During the study period 1,291 ICU patients were treated with RRT for AKI. Mortality was 47.2% at day 30 and 57.2% at hospital discharge. Mortality in hospital survivors showed an important increase until 3-year follow-up, and a moderate increase later (1 year: 14.4%, 2 years: 20%, 3 years: 35.7%, and 7 years: 39%). In-hospital survivors' Scr and eGFR at baseline were comparable to 1-year follow-up (1.4 vs. 1.4 mg/dl, *P *= 0.162, respectively 46 vs. 51 ml/minute/1.73 m^2^) and we observed an increase of CKD stage in 36.0% of patients, a decrease in 36.3%, and stable CKD stage in 27.7%. A total 43.1% of patients with CKD stage <3 at baseline had an increase to CKD stage ≥3 at 1 year. A total 26.6% of patients with CKD stage ≥3 at baseline decreased to CKD stage <3. A total of 8.3% of hospital survivors developed ESKD. Patients with increase of CKD stage had similar age (67 vs. 64 years, *P *= 0.145), SAPS 2 (57 vs. 51, *P *= 0.858), and SOFA score (9 vs. 9, *P *= 0.275) compared with patients with stable or decreased CKD stage. There were no differences in type of ICU, modality of RRT, or number of patients treated with vasopressors (44% vs. 47%, *P *= 0.617) or invasive ventilation (84% vs. 81%, *P *= 0.496). However, these patients were more probably male (76.9% vs. 65.6%, *P *= 0.042), had lower Scr at baseline (1.1 vs. 1.7 mg/dl, *P *< 0.001) and at ICU admission (1.7 vs. 2.4 mg/dl, *P *< 0.001), and were started on RRT later (3 vs. 2 days, *P *= 0.008).

## Conclusion

The annual mortality in AKI-RRT hospital survivors is approximately 10% per year during the first 3 years of follow-up. One- third of patients had an increase of CKD stage at 1 year of follow-up and almost one-half of patients who had eGFR >60 ml/minute/1.73 m^2 ^developed CKD. Patients who had an increase of CKD had similar severity of illness, but lower Scr at baseline and at ICU admission, and had later initiation of RRT compared with patients who had stable or decreased CKD stage.

